# The Extract of Roots of *Sophora flavescens* Enhances the Recovery of Motor Function by Axonal Growth in Mice with a Spinal Cord Injury

**DOI:** 10.3389/fphar.2015.00326

**Published:** 2016-01-14

**Authors:** Norio Tanabe, Tomoharu Kuboyama, Kohei Kazuma, Katsuhiro Konno, Chihiro Tohda

**Affiliations:** ^1^Division of Neuromedical Science, Department of Bioscience, Institute of Natural Medicine, University of ToyamaToyama, Japan; ^2^Division of Kampo-Pharmaceutics, Department of Medical Resources, Institute of Natural Medicine, University of ToyamaToyama, Japan

**Keywords:** *Sophora flavescens*, matrine, oxymatrine, axonal growth, chondroitin sulfate proteoglycan, spinal cord injury

## Abstract

Although axonal extension to reconstruct spinal tracts should be effective for restoring function after spinal cord injury (SCI), chondroitin sulfate proteoglycan (CSPG) levels increase at spinal cord lesion sites, and inhibit axonal regrowth. In this study, we found that the water extract of roots of *Sophora flavescens* extended the axons of mouse cortical neurons, even on a CSPG-coated surface. Consecutive oral administrations of *S. flavescens* extract to SCI mice for 31 days increased the density of 5-HT-positive axons at the lesion site and improved the motor function. Further, the active constituents in the *S. flavescens* extract were identified. The water and alkaloid fractions of the *S. flavescens* extract each exhibited axonal extension activity *in vitro*. LC/MS analysis revealed that these fractions mainly contain matrine and/or oxymatrine, which are well-known major compounds in *S. flavescens*. Matrine and oxymatrine promoted axonal extension on the CSPG-coated surface. This study is the first to demonstrate that *S. flavescens* extract, matrine, and oxymatrine enhance axonal growth *in vitro*, even on a CSPG-coated surface, and that *S. flavescens* extract improves motor function and increases axonal density in SCI mice.

## Introduction

People who have suffered spinal cord injury (SCI) have serious motor dysfunction and abnormal sensation due to the disruption of the descending motor and ascending sensory tracts at the lesion site. Although no effective drug therapy currently exists, the reconstruction of spinal tracts by axonal growth is the most effective strategy for regaining function. At the lesion site, reactive astrocytes produce inhibitory extracellular matrix molecules, such as chondroitin sulfate proteoglycan (CSPG; McKeon et al., 1999; Tang et al., 2003). Because the degradation of CSPG by chondroitinase ABC (ChABC) led to functional recovery in SCI rats (Bradbury et al., 2002), CSPG has received attention as a major molecule that inhibits axonal growth. This approach utilizes intrinsic axonal growth activity. In contrast, we aimed to look for strong axonal growth activity that is not defeated by CSPG. To find this activity, we previously performed a CSPG coating assay and screened water extracts of 110 types of crude drugs that have been used in traditional Japanese medicine. In this assay, the extract of dried roots of *Sophora flavescens* Aiton (Leguminosae) exhibited remarkable capability to stimulate axonal growth on a CSPG coating.

The dried roots of *S. flavescens*, Sophorae Radix, is an anti-febrile, diuretic, and anti-parasitic agent (Commission of Chinese Pharmacopoeia, 2010) and has been used traditionally in many countries, including China, Korea, and Japan. In this study, new therapeutic potential of dried roots of *S. flavescens* was investigated in SCI mice.

## Materials and methods

All experiments were performed in accordance with the Guidelines for the Care and Use of Laboratory Animals of the University of Toyama. The committee for Animal Care and Use at the Sugitani Campus of the University of Toyama approved the study protocols. The approval number for the animal experiments is A2013INM-1. All efforts were made to minimize the number of animals used.

### Preparation of extracts and fractions

Dried roots of *S. flavescens*, which were collected from Hebei Province, Guizhou Province and Shanxi Province, China, were purchased from Tochimoto Tenkaido (Osaka, Japan; a voucher specimen NMS No. 002612001). Dried roots of *S. flavescens* (280 g) were heated with water in a 20:1 water:root ratio (5.6 L water) at 100°C for 1 h. After filtration with a pledget, the extract was freeze-dried to obtain a powdered extract (43 g; yield was 15.4%).

To obtain subfractions, *S. flavescens* roots (45 g) were extracted under the same condition mentioned above. After filtration, a portion of the extract (300 ml) was partitioned twice with ethyl acetate (100 ml). The water layer was mixed with ammonia and partitioned twice with chloroform (100 ml) to obtain an alkaloid fraction. These extracts were ultimately obtained as powders (57 mg for EtOAc fr., 425 mg for alkaloid fr., and 2411 mg for H_2_O fr.).

### Primary culture and CSPG coating assay

Eight-well chamber slides (Falcon, Franklin Lakes, NJ, USA) were coated with 5 μg/ml poly-d-lysine (PDL; Sigma-Aldrich, St. Louis, MO, USA) overnight at 37°C. For the CSPG coating assay, 2.0 μg/ml aggrecan (Sigma-Aldrich), a CSPG, or vehicle solution was further applied to the PDL-coated slide for 5 h at 37°C. Primary cultured cortical cells were obtained from E14 embryos of ddY mice (Japan SLC, Shizuoka, Japan) as previously described (Watari et al., 2014). The cells were cultured on eight-well chamber slides with Neurobasal medium (Life Technologies, Carlsbad, CA, USA) containing 12% horse serum, 0.6% d-glucose, and 2 mM l-glutamine and maintained at 37°C in a humidified incubator at 10% CO_2_. Five hours after the culture started, the medium was replaced with fresh Neurobasal medium containing 2% B-27 supplement without horse serum. On the next day of the culture, the *S. flavescens* extract (1 and 10 μg/ml), ethyl acetate fraction (1 and 10 μg/ml), alkaloid fraction (1 and 10 μg/ml), water fraction (1 and 10 μg/ml), matrine (1 and 10 μM; Tokyo Chemical Industry Co., Tokyo, Japan), oxymatrine (1 and 10 μM; Santa Cruz Biotechnology Inc., Dallas, TX, USA), or vehicle solution was applied to the cells. Four days after the treatment, the cells were fixed with 4% paraformaldehyde and immunostained with mouse anti-phosphorylated neurofilament-H (pNF-H, a marker of axons) monoclonal antibody (clone, SMI-35; dilution, 1:500; Covance, Princeton, NJ, USA) and rabbit anti-microtubule associated protein 2 (MAP2, a marker of neuronal cell bodies) polyclonal antibody (dilution, 1:2000; Abcam, Cambridge, UK). The secondary antibodies were Alexa Fluor 488-conjugated goat anti-rabbit IgG (dilution, 1:400; Life Technologies) and Alexa Fluor 594-conjugated goat anti-mouse IgG (dilution, 1:400; Life Technologies). The cells were counterstained with 4′,6-diamidino-2-phenylindole (DAPI, a marker of nuclei; 0.1 μg/ml; Enzo Life Science, Farmingdale, NY, USA). Fluorescence images were acquired at a size of 670 × 890 μm using a BX61/DP70 microscope (Olympus, Tokyo, Japan). On each image, the total length of the axons was automatically measured using a Neurocyte image analyzer (Kurabo, Osaka, Japan), and the number of cell bodies was manually counted to calculate the axonal density per neuron.

### SCI operation

All mice were housed with *ad libitum* access to food and water and were kept in a constant environment (22 ± 2°C, 50 ± 5% humidity, 12-h light cycle starting at 07:00). Eight-week-old female ddY mice (SLC) were used for SCI experiments. The mice were laminectomized under anesthesia with trichloroacetaldehyde monohydrate (450–500 mg/kg, i.p.). A 6.5-g weight was dropped from a height of 2 cm onto the exposed spinal cord at T9-10 level using a stereotaxic instrument (Narishige, Tokyo, Japan) to produce a contusion injury. *S. flavescens* extract (500 mg/kg/day) or a vehicle control was continually administered to SCI mice from 1 h after the injury for 31 days once a day. The hindlimb motor functions of SCI mice were evaluated using the Basso Mouse Scale (BMS; Basso et al., 2006), Body Support Scale (BSS; Teshigawara et al., 2013), and Toyama Mouse Score (TMS; Shigyo et al., 2014) in an open field (black color, 50.0 × 42.5 × 15.0 cm) under 500-lux illumination.

### Immunohistochemistry

Thirty-one days after the SCI, the mice were anesthetized by administration of trichloroacetaldehyde monohydrate and were transcardially perfused with ice-cold saline and 4% paraformaldehyde. Spinal cord tissues around the injured region were removed and soaked in 4% paraformaldehyde and then solutions of increasing sucrose concentration (10, 20, and 30%). The spinal cord tissues were cut into 12-μm successive sagittal sections using a CM3050S cryostat (Leica, Heidelberg, Germany). The slices were fixed with 4% paraformaldehyde and immunostained with rabbit anti-5-hydroxytryptamine (5-HT, a marker of the raphespinal tract) polyclonal antibody (dilution 1:500; ImmunoStar, Hudson, Wisconsin, USA), mouse anti- glial fibrillary acidic protein (GFAP, a marker of reactive astrocytes) monoclonal antibody (clone, G-A-5; dilution 1:1000; Sigma-Aldrich), and mouse anti-CSPG monoclonal antibody (clone, CS-56; dilution 1:500; Sigma-Aldrich). The secondary antibodies were Alexa Fluor 488-conjugated goat anti-rabbit IgG (dilution, 1:200; Life Technologies), Alexa Fluor 594-conjugated goat anti-mouse IgG_1_ (dilution, 1:400; Life Technologies), and Alexa Fluor 350-conjugated goat anti-mouse IgM (dilution, 1:400; Life Technologies). Fluorescence images were acquired using an Axio Observer Z1 microscopy system (Carl Zeiss, Oberkochen, Germany) and tiled. Lesion sites were identified based on the fluorescence images of GFAP. The sizes of the lesion site, expression levels of CSPG inside the lesion site and densities of the 5-HT positive area inside the lesion site were quantified using ImageJ image analysis software (NIH).

### LC/MS analysis of the alkaloid fraction, the water fraction, matrine, and oxymatrine

The alkaloid fraction, the water fraction, matrine, and oxymatrine were analyzed using a Thermo Scientific LC/MS system (Thermo Fisher Scientific, Waltham, Massachusetts, USA), which consisted of an Accella-600 HPLC system with photodiode array detection and an LTQ Orbitrap XL Mass Spectrometer. The LC/MS analysis conditions were as follows. The HPLC conditions: solvent A, 0.1% formic acid in H_2_O; solvent B, 0.1% formic acid in MeCN; column, Capcell Pak C-18 (1.5 mm i.d. × 150 mm; Shiseido, Tokyo, Japan); flow, 0.20 ml/min; gradient program, a linear gradient of 2–35% of solvent B in solvent A for 15 min followed by a linear gradient of 35–90% of solvent B for 5 min, and then maintenance of 90% solvent B in solvent A for 5 min; column temp., 25°C; and photodiode array detection, 200–600 nm. The MS conditions: electrospray polarity, positive mode; sheath gas flow, 50 arbitrary units; aux gas flow, 30 arbitrary units; ion spray voltage, 4.6 kV; capillary temp., 350°C; capillary voltage 19.0 V; tube lens, 35 V; and detector, an Orbitrap detector with a high resolution of 60,000.

### Statistical analysis

Statistical comparisons were performed through One-way analysis of variance (ANOVA) with the *post hoc* Bonferroni test, repeated measures Two-way ANOVA with *post hoc* Bonferroni test, or an unpaired two-tailed *t*-test using GraphPad Prism 5 (GraphPad Software, San Diego, California, USA). *P* < 0.05 was considered significant. The data are presented as the mean ± SE.

## Results

### *S. flavescens* extract promotes axonal extension on a CSPG-coated surface

**Figure 1:**
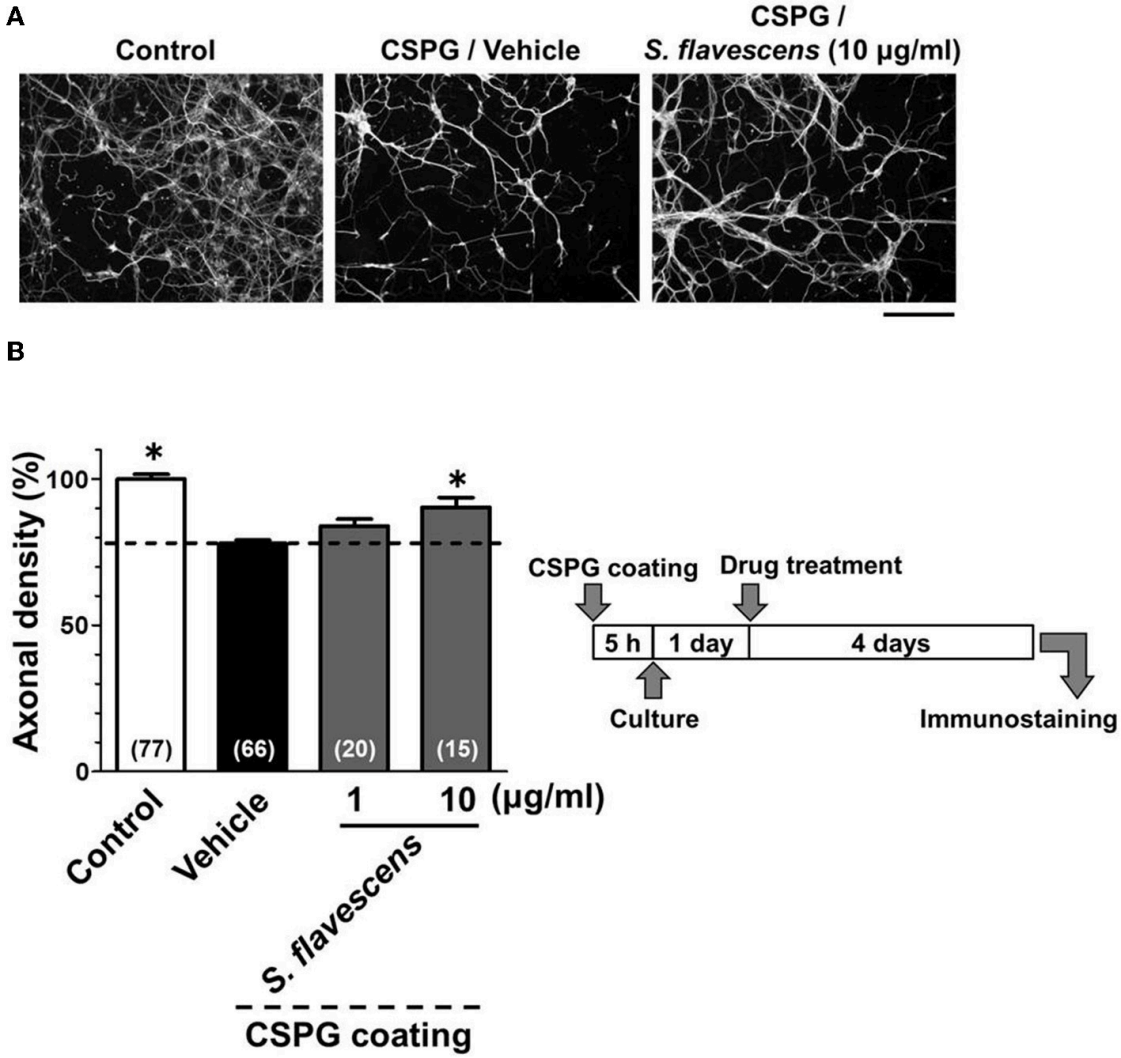
*****S. flavescens*** extract promotes axonal extension on the CSPG coating**. Cortical neurons were cultured for 1 day and then treated with *S. flavescens* extract (1 and 10 μg/ml) or vehicle solution. Four days after treatment, the cells were fixed and immunostained for pNF-H and MAP2. **(A)** Representative immunofluorescence images of pNF-H for each treatment. **(B)** The densities of pNF-H-positive axons per neuron were quantified for each treatment. The numbers in parentheses indicate the measured number of captured images. ^*^p < 0.05 vs. CSPG coating/vehicle-treated group, One-way ANOVA *post hoc* Bonferroni test. Scale bar = 200 μm.

For the CSPG coating assay, culture slides were coated with PDL and then with CSPG. Axonal density was significantly decreased on the CSPG substrate compared with that on the PDL substrate only (Figures 1A,B). *S. flavescens* extract (10 μg/ml) treatment significantly increased the axonal density on the CSPG substrate compared with that for the vehicle treatment. The enhancement of axonal extension by the *S. flavescens* extract was not observed under normal coating (**Figure 7**).

### *S. flavescens* extract increases axons at the lesion site and improves motor dysfunction in SCI mice

**Figure 2:**
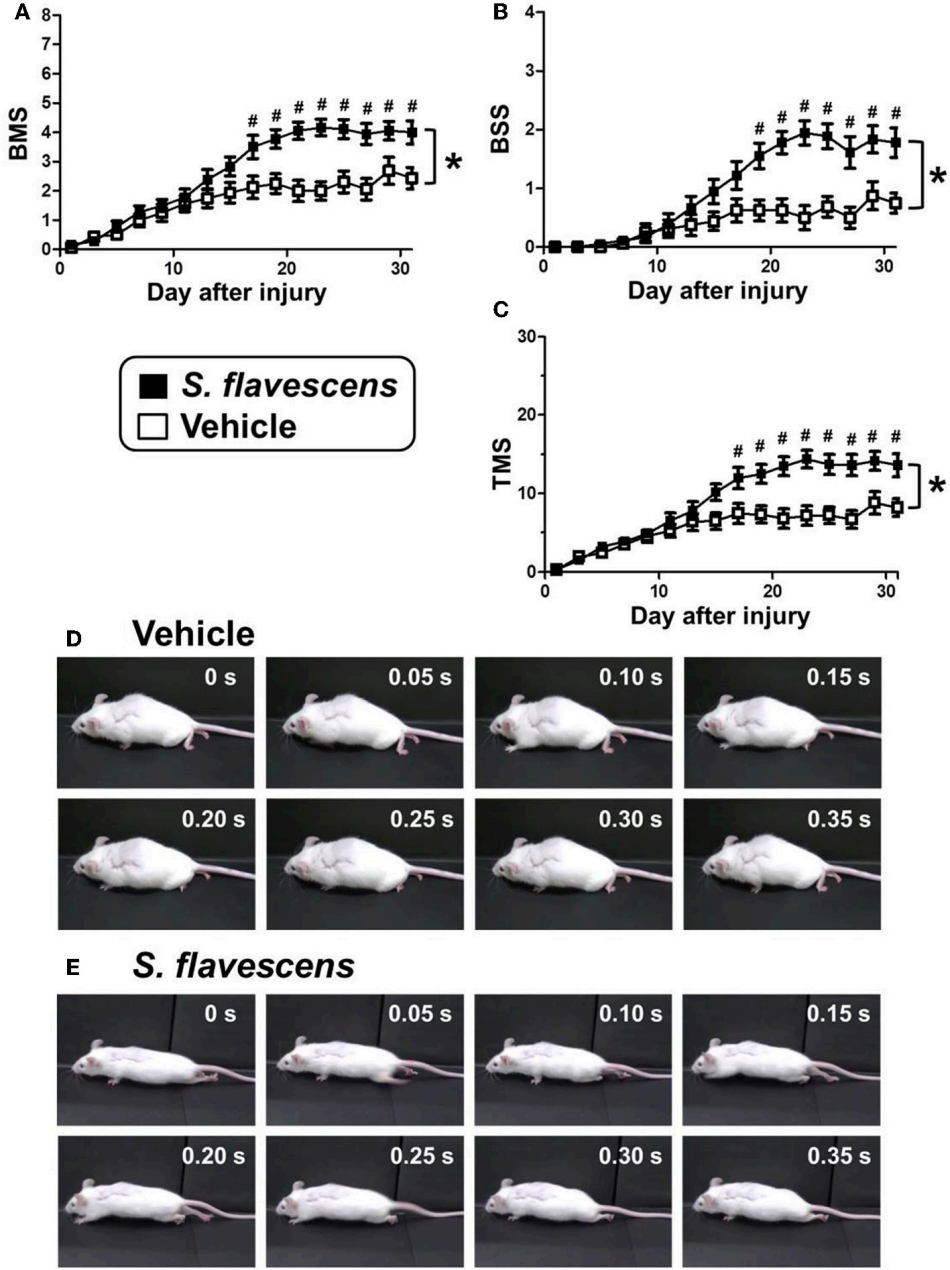
*****S. flavescens*** extract improves motor dysfunction in SCI mice**. SCI mice were administered *S. flavescens* extract (black squares, nine mice, 18 hindlimbs, *n* = 18) or vehicle solution (white squares, eight mice, 16 hindlimbs, *n* = 16) once a day from 1 h after the injury for 31 days. The motor function of hindlimbs was assessed using BMS **(A)**, BSS **(B)**, and TMS **(C)**. At 31 days after SCI, the ambulation of a representative SCI mouse treated with vehicle solution **(D)** or *S. flavescens* extract **(E)** was captured. Sequentially captured images obtained every 0.05 s are shown. ^*^p < 0.0001, time × drug interaction analyzed by repeated measures Two-way ANOVA. #*p* < 0.05, *post hoc* Bonferroni test.

To investigate the effects of *S. flavescens* extract in SCI mice, *S. flavescens* extract (500 mg/kg/day), or vehicle solution was orally administered to SCI mice. The drug administration was started from 1 h after the SCI operation and was continued for 31 days. The hindlimb motor functions were assessed using BMS, BSS, and TMS for 31 days, once every 2 days. For all of the evaluation methods, repeated measures Two-way ANOVA revealed a significant time × drug interaction between the vehicle group and the *S. flavescens* extract group [*F*_(15, 480)_ = 9.434, *p* < 0.0001 (BMS; Figure 2A); *F*_(15, 480)_ = 9.397, *p* < 0.0001 (BSS; Figure 2B); *F*_(15, 480)_ = 10.68, *p* < 0.0001 (TMS; Figure 2C)]. The scores of *S. flavescens* extract-treated mice were significantly higher than those of vehicle-treated mice from 17 to 31 days after the injury for the BMS and TMS evaluations and from 19 to 31 days after the injury for the BSS evaluation. At 31 days after the injury, vehicle-treated mice had scores of 2.44 in BMS, 0.75 in BSS, and 8.19 in TMS. These scores show that the mice could not touch the floor with the soles of their feet (Figure 2D and Supplementary Video 1). In contrast, *S. flavescens* extract-treated mice had scores of 4.00 in BMS, 1.78 in BSS, and 13.6 in TMS, showing that the mice could touch the floor with the soles of their feet and sometimes could lift up the hindquarters (Figure 2E, especially the photos at 0.15 and 0.20 s, and Supplementary Video 2).

**Figure 3:**
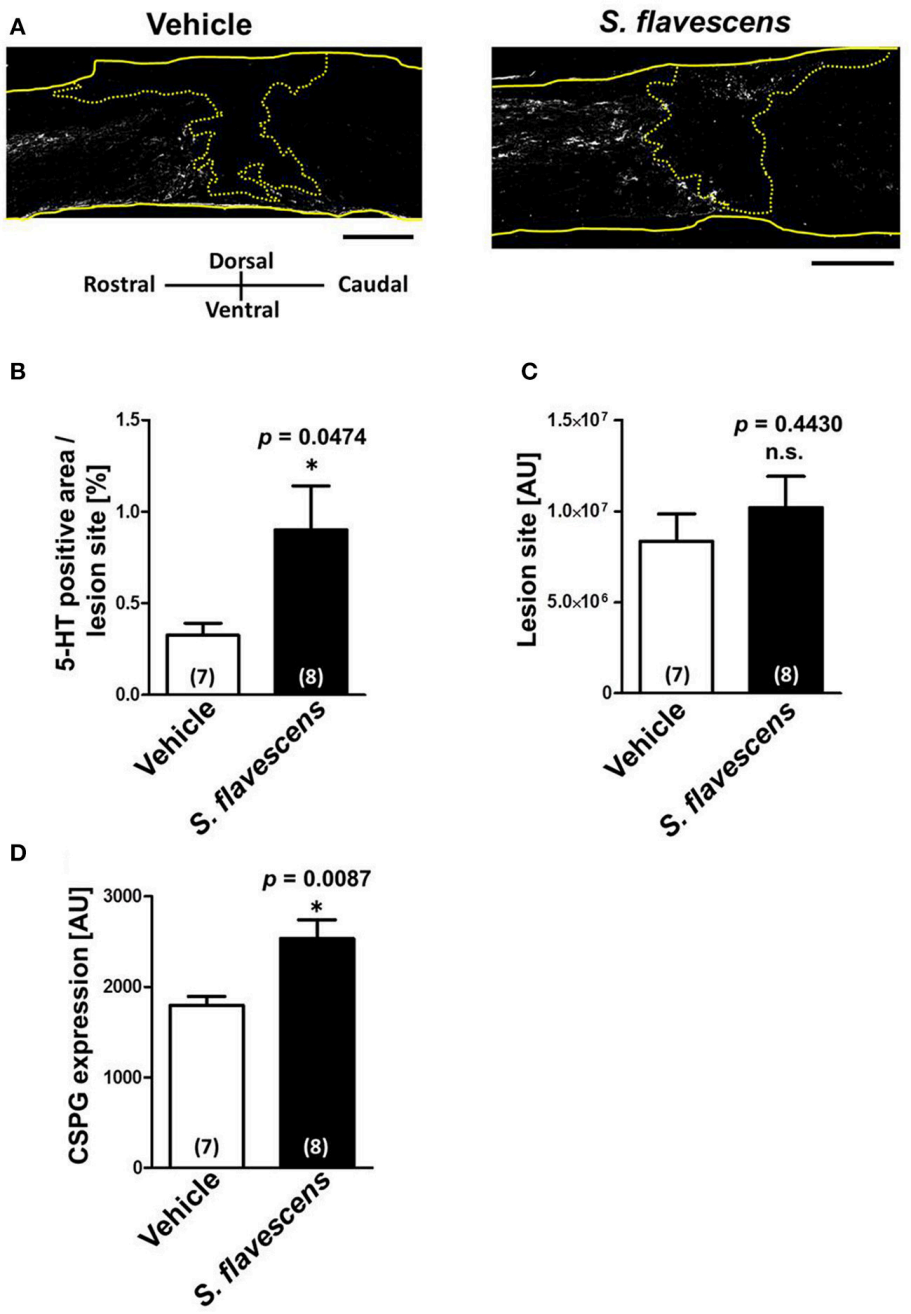
*****S. flavescens*** extract increases the axon density at lesion sites in SCI mice**. Sagittal sections of spinal cords were obtained from *S. flavescens* extract—or vehicle-treated SCI mice at 31 days after the injury. The sections were immunostained for 5-HT, GFAP, and CSPG. **(A)** Representative immunofluorescence images of 5-HT. The yellow solid lines indicate outlines of the spinal cords. The yellow dotted line surrounds the lesion sites identified using the immunofluorescence images of GFAP. **(B–D)** The 5-HT-positive area at the lesion site **(B)**, size of the lesion site **(C)**, and expression of CSPG at the lesion site **(D)** were quantified. The numbers in parentheses indicate the numbers of mice. ^*^p < 0.05 vs. vehicle group, unpaired two-tailed *t*-test. Scale bar = 500 μm.

To confirm whether axonal extensions at the lesion site were induced by *S. flavescens* extract treatment, spinal cords were isolated at 31 days after the injury. Spinal cord slices were immunostained for 5-HT (Figure 3A), GFAP, and CSPG. The molecule 5-HT is a marker of serotonergic raphespinal tracts, one of the major tracts that regulates motor function (Liu and Jordan, 2005). GFAP is a marker of reactive astrocytes. The lesion site was defined as the area surrounded by GFAP-positive cells. The *S. flavescens* extract showed no effect on the size of the lesion site (Figure 3C). The density of the 5-HT-positive area at the lesion site was significantly increased in the *S. flavescens* extract-treated mice (Figures 3A,B). However, the *S. flavescens* extract-treated group showed significantly upregulated expression levels of CSPG at the lesion site (Figure 3D). These results suggest that *S. flavescens* extract promotes the axonal extension of at least raphespinal tracts, even in the presence of CSPG *in vivo*, and the axonal extension by the *S. flavescens* extract does not seem to be blocked by the *S. flavescens* extract-induced CSPG increase.

### Matrine and oxymatrine are active constituents in *S. flavescens* extract for the axonal extension activity on a CSPG-coated surface

**Figure 4:**
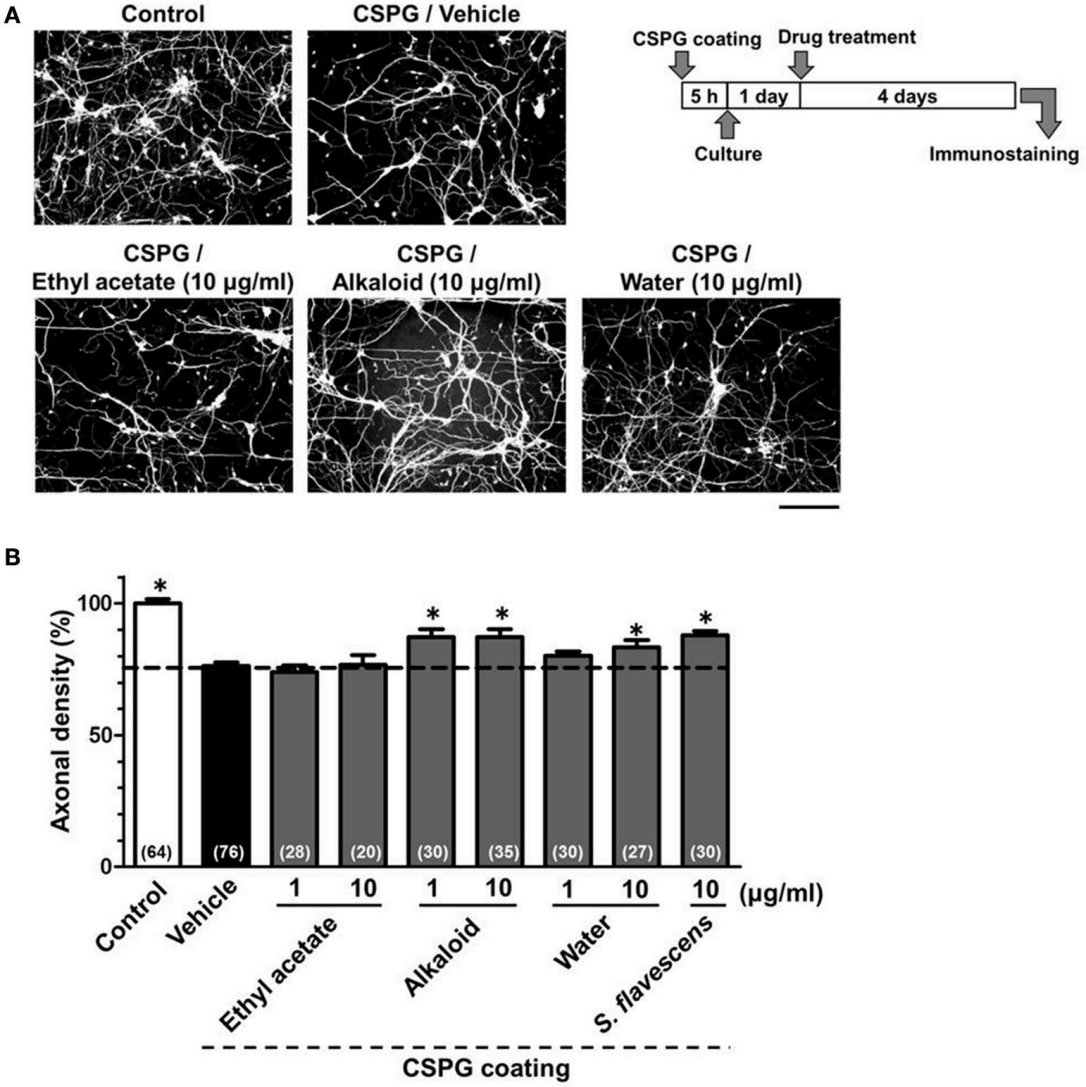
**The alkaloid fraction and the water fraction promote axonal extension on the CSPG coating**. Cortical neurons were cultured for 1 day and then treated with the ethyl acetate fraction (1 and 10 μg/ml), the alkaloid fraction (1 and 10 μg/ml), the water fraction (1 and 10 μg/ml), *S. flavescens* extract (10 μg/ml), or vehicle solution. Four days after treatment, the cells were fixed and immunostained for pNF-H and MAP2. **(A)** Representative immunofluorescence images of pNF-H for each treatment. **(B)** The densities of pNF-H-positive axons per neuron were quantified for each treatment. The numbers in parentheses indicate the measured number of captured images. ^*^p < 0.05 vs. CSPG coating/vehicle-treated group, One-way ANOVA *post hoc* Bonferroni test. Scale bar = 200 μm.

**Figure 5:**
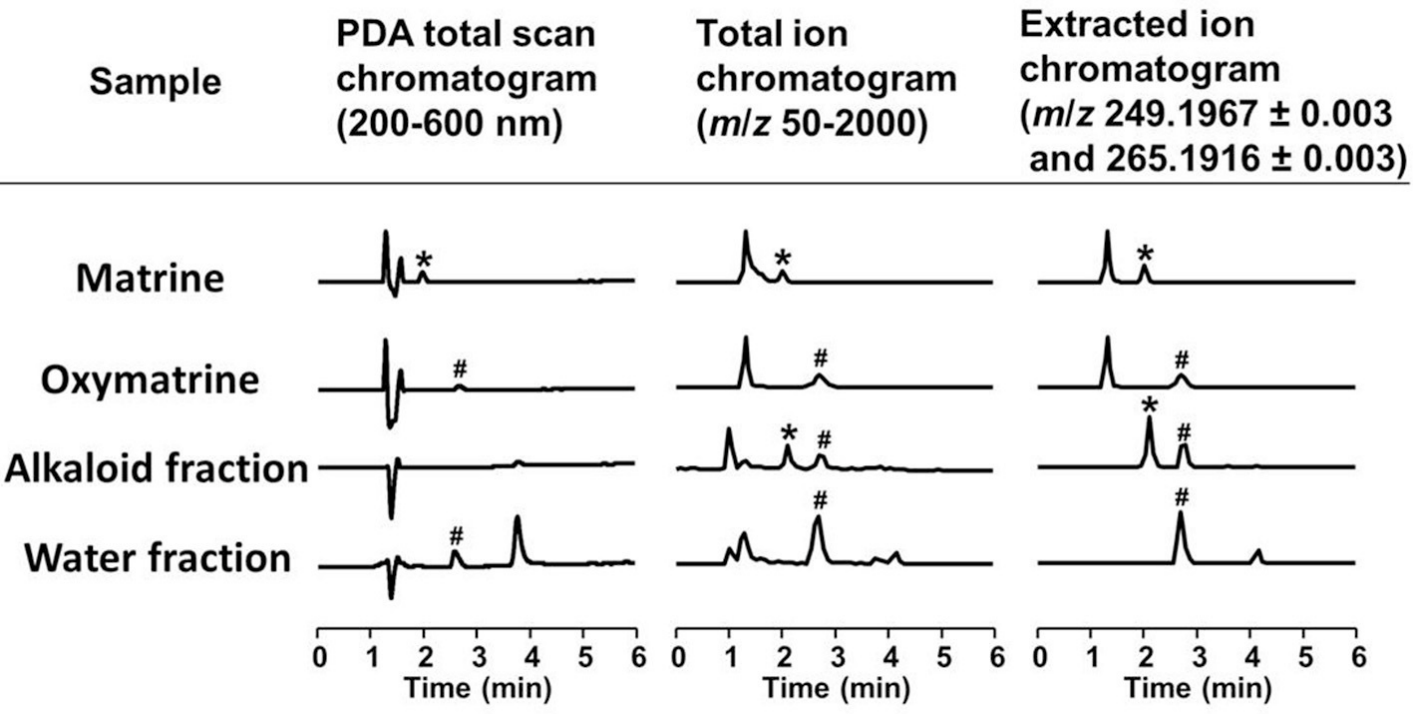
**LC/MS analysis of the alkaloid and water fractions**. Photodiode array (PDA) total scan chromatograms, total ion chromatograms, and extracted ion chromatograms at *m/z* 249.1967 ± 0.003 and 265.1916 ± 0.003 to detect matrine and oxymatrine, respectively, in the alkaloid fraction and the water fraction. ^*^A specific peak of matrine; ^#^a specific peak of oxymatrine.

**Figure 6:**
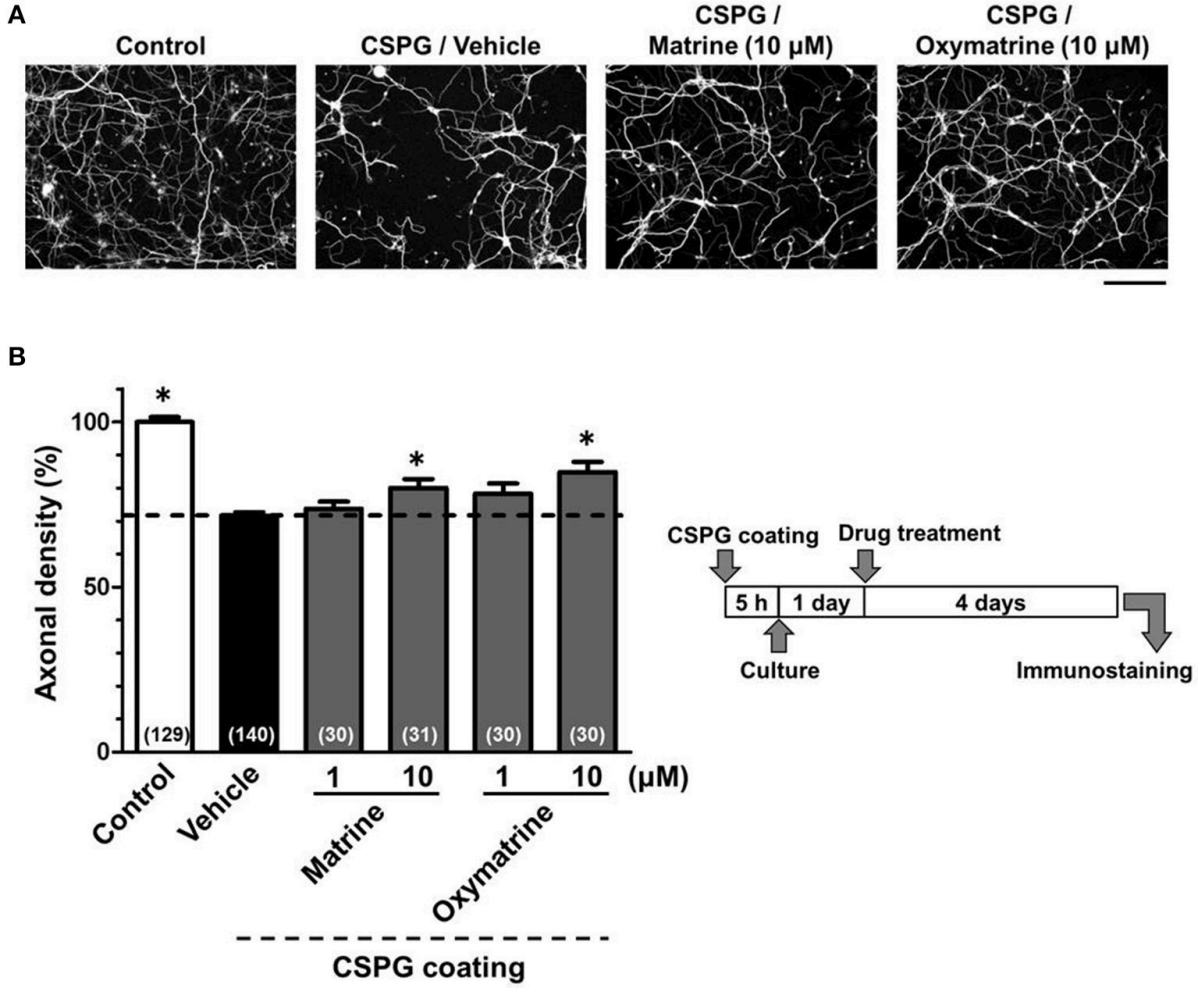
**Matrine and oxymatrine promote axonal extension on the CSPG coating**. Cortical neurons were cultured for 1 day and then treated with matrine (1 and 10 μM), oxymatrine (1 and 10 μM), or vehicle solution. Four days after the treatment, the cells were fixed and immunostained for pNF-H and MAP2. **(A)** Representative immunofluorescence images of pNF-H for each treatment. **(B)** The density of pNF-H-positive axons per neuron were quantified for each treatment. The numbers in parentheses indicate the measured number of captured images. ^*^p < 0.05 vs. CSPG coating/vehicle-treated group, One-way ANOVA *post hoc* Bonferroni test. Scale bar = 200 μm.

**Figure 7:**
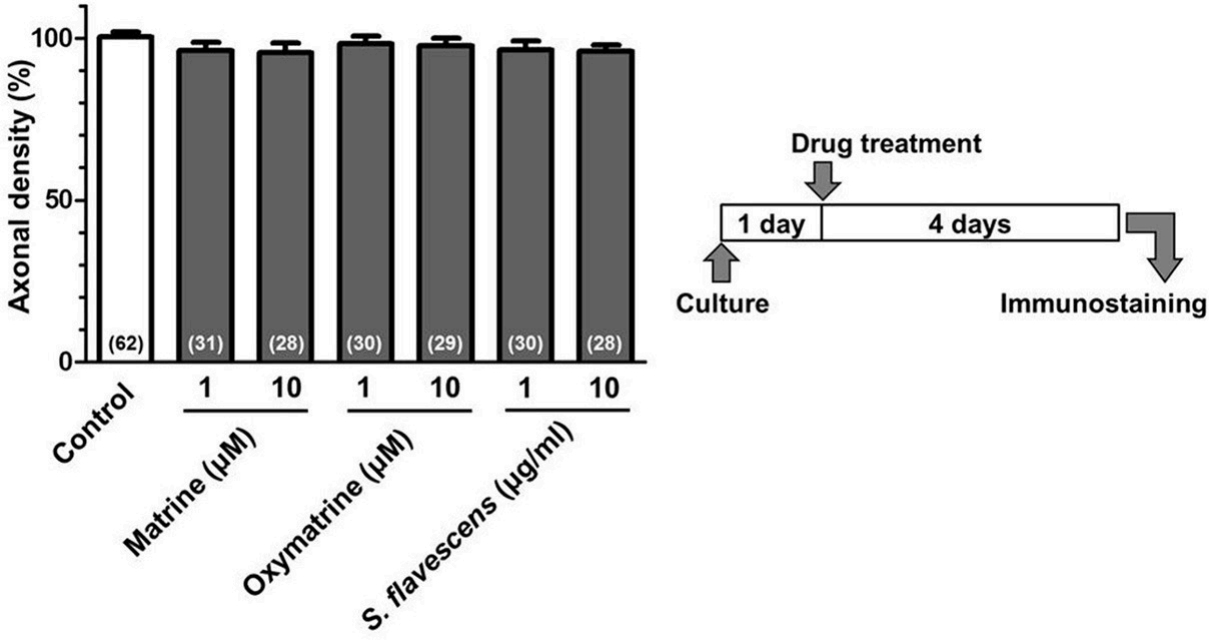
**Matrine, oxymatrine, and ***S. flavescens*** extract have no effects on axonal extension on the PDL coating**. Cortical neurons were cultured for 1 day and then treated with matrine (1 and 10 μM), oxymatrine (1 and 10 μM), *S. flavescens* extract (1 and 10 μg/ml), or vehicle solution. Four days after the treatment, the cells were fixed and immunostained for pNF-H and MAP2. The density of pNF-H-positive axons per neuron were quantified for each treatment. The numbers in parentheses indicate the measured number of captured images.

To identify the active constituents in the *S. flavescens* extract, the *S. flavescens* extract was separated into ethyl acetate, alkaloid, and water fractions. These fractions (1 and 10 μg/ml) were evaluated for axonal extension activity using the CSPG coating assay (Figure 4). Compared with vehicle solution, the alkaloid fraction (1 and 10 μg/ml), and the water fraction (10 μg/ml) significantly promoted axonal extension on a CSPG-coated surface, but the ethyl acetate fraction did not exhibit these effects. This result suggested that the active constituents in *S. flavescens* extract were contained in the alkaloid fraction and the water fraction, but not in the ethyl acetate fraction. Previous study by Yang et al. showed that alkaloids were rich in a water layer, and flavonoids were rich in an ethyl acetate layer when the extract of *S. flavescens* was partitioned with water and ethyl acetate (Yang et al., 2012). We determined whether the alkaloid and water fractions contained matrine and oxymatrine using LC/MS analysis (Figure 5) because matrine and oxymatrine are major alkaloids in *S. flavescen*s (Li and Wang, 2004). Specific peaks of matrine and oxymatrine were detected at retention times of 2 min and 2 min 40 s, respectively. The peak of matrine was detected in the alkaloid fraction, and the peak of oxymatrine was detected in both the alkaloid and water fractions. Based on MS analysis, the molecular weights of these peaks corresponded to matrine (*m*/*z*: 249.1967 ± 0.003) and oxymatrine (*m*/*z*: 265.1916 ± 0.003). These results suggested that the alkaloid fraction contained substantial amount of matrine and oxymatrine and that the water fraction contained an abundance of oxymatrine. Then, the effects of matrine (1 and 10 μM) and oxymatrine (1 and 10 μM) on axonal extension were evaluated using a CSPG coating assay (Figure 6). Compared with vehicle solution, matrine (10 μM), and oxymatrine (10 μM) significantly increased the axonal density on the CSPG. When neurons were cultured on a normal PDL substrate without CSPG, matrine (1 and 10 μM), and oxymatrine (1 and 10 μM) induced no axonal growth (Figure 7).

## Discussion

Recent studies showed several biological effects of *S. flavescens* extract, such as anti-cancer (Xiao et al., 2013), anti-arrhythmic (Kim et al., 2013), anti-allergic (Dev et al., 2011), anti-inflammatory (Kim et al., 2012), and anti-asthmatic effects (Yang et al., 2013). In particular, the anti-cancer effect has been actively investigated for *S. flavescens* extract and for several *S. flavescens* constituents, including matrine, and oxymatrine (Ren et al., 2014; Wu et al., 2015). Concerning the effects on the neuronal function, matrine exhibited protective effects on neurons and astrocytes under the conditions of oxygen- and glucose-deprivation, possibly through the inhibition of nuclear factor-κ B activation (Xu et al., 2012). In a rat model of experimental autoimmune encephalomyelitis, the administration of matrine suppressed the reduction of nerve fibers in the brain, which might relate to the increased level of brain-derived neurotrophic factor mRNA (Kan et al., 2015). Oxymatrine provides protective effects to neurons exposed to excessive n-methyl-d-aspartate (NMDA) via decreases in the up-regulation of NR2B-containing NMDA receptors, thereby suppressing the rapid elevation of the intracellular Ca^2+^ concentration (Zhang et al., 2013). In this study, we found that *S. flavescens* extract, matrine and oxymatrine extended axons, even on a CSPG-coated surface, and that *S. flavescens* extract administration ameliorated the motor dysfunction and increased the axonal density in SCI mice. These data are all new findings.

Although the CSPG expression at the lesion site in SCI mice was enhanced by *S. flavescens* extract administration (Figure 3D), the mechanism of the *S. flavescens* treatment-induced CSPG increase, and the constituents related to the phenomenon are unclear. Despite the CSPG elevation, axonal extension, and recovery of motor function were observed in the *S. flavescens* extract-treated SCI mice, suggesting that the axonal extension activity of *S. flavescens* extract may overcome the presence of CSPG. Although functional recovery in response to *S. flavescens* extract treatment in SCI mice may not be explained completely by the growth of 5-HT-positive raphespinal tracts, extension of the raphespinal tracts relates well to the improvement in motor function (Kim et al., 2004; Teshigawara et al., 2013; Bartus et al., 2014).

At least matrine and oxymatrine are a part of active constituents in the *S. flavescens* extract in this study. The mechanisms relating matrine and oxymatrine to axonal growth remain unknown, although antagonistic activities of those compounds on epidermal growth factor receptor (EGFR) were shown in relation to anti-cancer effects (Wang et al., 2010; Guo et al., 2015). Interestingly, an EGFR inhibitor promotes the regeneration of axonal fibers by blocking CSPG signals (Koprivica et al., 2005). Considering those results, the inhibition of EGFR signaling may be one possible pathway for the effects of matrine and oxymatrine. We are now comprehensively investigating the direct protein targets of matrine and oxymatrine. In addition, the effects of matrine and oxymatrine on motor dysfunction in SCI mice are also under investigation.

## Conclusion

This study is the first to demonstrate that *S. flavescens* extract, matrine and oxymatrine enhance axonal growth *in vitro*, even on a CSPG-coated surface, and that *S. flavescens* extract improves motor function and increases axonal density in SCI mice. These findings may lead to the development of a new SCI therapy.

## Funding

This work was supported by a Grant-in-Aid for challenging Exploratory Research No. (26670044) from the Ministry of Education, Culture, Sports, Science, and Technology of Japan (CT) and a Grant-in-Aid for The Cooperative Research Project from Institute of Natural Medicine, University of Toyama in 2014 and 2015 (CT and TK).

### Conflict of interest statement

The authors declare that the research was conducted in the absence of any commercial or financial relationships that could be construed as a potential conflict of interest.
