# Supplementary material for: The Extract of Roots of Sophora flavescens Enhances the Recovery of Motor Function by Axonal Growth in Mice with a Spinal Cord Injury
Source: Front Pharmacol. 2016 Jan 14;6:326. doi: 10.3389/fphar.2015.00326 (PMC4712302; doi:10.3389/fphar.2015.00326)
Supplement: Supplementary file 3 [file DataSheet1.DOCX]

Supplementary Material

The extract of roots of *Sophora flavescens* enhances the recovery of motor function by axonal growth in mice with a spinal cord injury

**Norio Tanabe, Tomoharu Kuboyama, Kohei Kazuma, Katsuhiro Konno, Chihiro Tohda ***

*** Correspondence:** Chihiro Tohda, Ph.D.: chihiro@inm.u-toyama.ac.jp.

# 1. Supplementary Video

**Supplementary Video 1.** The ambulation of a representative vehicle solution-treated SCI mouse at 31 days after SCI

**Supplementary Video 2.** The ambulation of a representative *S. flavescens* extract-treated SCI mouse at 31 days after SCI
